# Videogames and Therapy: A Narrative Review of Recent Publication and Application to Treatment

**DOI:** 10.3389/fpsyg.2016.01085

**Published:** 2016-07-14

**Authors:** Gilbert E. Franco

**Affiliations:** ^1^Southern California Seminary, Graduate Behavioral SciencesEl Cajon, CA, USA; ^2^College of Social Sciences, University of PhoenixSan Diego, CA, USA

**Keywords:** videogames, video games, psychotherapy, counseling, psychology, group therapy, group processes

## Abstract

Individuals who play videogames can interact with virtual worlds, resulting in emotional and intellectual connections that have therapeutic implications in the hands of a skilled and informed therapist. There is research available in the literature that suggests that videogames are a viable option in psychotherapy. The present article provides a review of the literature available in the use of videogames in treatment, discusses the importance of disseminating the findings in the literature, and discusses the integration of videogames in treatment.

The human mind is capable of tremendous feats. Its capacity for creation is seemingly boundless with timeless works of architecture, science, and art present in humankind's ever expanding history. The Roman Colosseum, Mona Lisa, and even the construction of modern airplanes serve as examples of the human potential to create. The modern advent of videogames takes human creativity and applies it to worlds that do not physically exist. People who play videogames can interact with virtual worlds, but this does not make the experience any less real for videogame players. Emotional and intellectual connections that people can make in videogames can have therapeutic implications in the hands of a skilled and informed therapist.

The relationship between videogames and human social, political, mental, physical, and even creative variables has been studied in the literature (e.g., Montani et al., 2014; Sil et al., 2014; Alhabash and Wise, 2015; Bloom, 2015; Bock et al., 2015; Chang et al., 2015; Yeh, 2015; González et al., 2016; Nebel et al., 2016). For example, Chang et al. (2015) looked at cognitive styles, levels of prior abilities, and how they influence learning achievement and frustration tolerance in a group-based videogame. They found that field independent students, which can differentiate between the details and the surrounding context when learning, are better than field dependent students, which cannot differentiate between details and the surrounding context, in learning achievement. On the other hand, field dependent students showed more frustration tolerance.

The differences between these groups can have implications for videogame-based group therapy because of these differences in learning styles. For example, field dependent clients may learn therapeutic concepts quickly using videogames that have social content and externally defined goals. Field independent clients may react better with the use of analytical puzzle games to stimulate their thoughts. Field independent students may react less favorably to videogame-based group therapy than field dependent clients. Figure 1 depicts the most frequently played videogames according to the Entertainment Software Association (2015).

**Figure 1:**
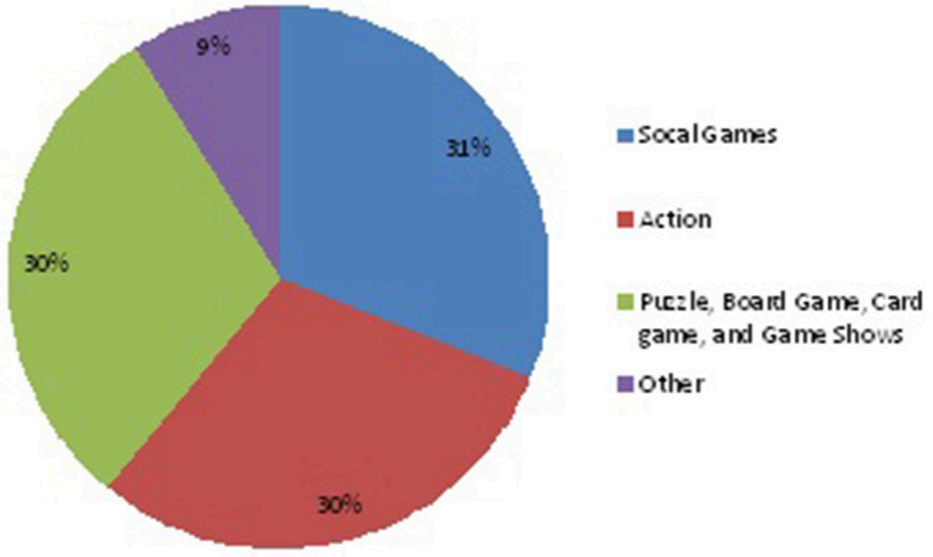
**Most frequently played voideogames reported by the (Entertainment Software Association, 2015)**.

Llorens et al. (2015) looked at videogame-based group therapy and its application to individuals suffering from traumatic brain injury (TBI). They conducted weekly 1 h videogame-based group therapy once a week for 6 months. They found that videogame integrative videogame-based group therapy are a motivating and effective treatment approach to improving self-awareness, social skills, and behaviors in people with TBI. In addition to using videogame-based treatment on those with TBI, videogames can be used with other diagnoses.

Fernandez-Aranda et al. (2015) investigated videogames as a therapeutic tool for cognitive behavioral therapy (CBT) in clients with bulimia nervosa. They looked at how CBT coupled with a serious videogame can address a client's emotional dysregulation. They found that clients with CBT, coupled with a serious videogame had less dropouts, more partial remission and total remission than the CBT control group. They concluded that CBT coupled with a serious videogame is a good tool that therapists can use to improve emotional dysregulation in clients with bulimia nervosa.

The results of the Llorens et al. (2015), Fernandez-Aranda et al. (2015) and Chang et al. (2015) studies shed some light as to the possibilities of using videogames in counseling and psychotherapy. Therapists can now consider using videogames as tools to use in treatment. Much like a therapist can use the empty chair technique to enable a client to gain insight or awareness and to bring them into the present; videogames can be used in treatment.

Videogames are part of a multibillion dollar industry (Franco, 2015). As a result, there is a good chance that many prospective clients play videogames. Table 1 depicts some demographical data obtained from the Entertainment Software Association (2015). At a very basic level, videogames can be used to build rapport with a client. For example, a therapist can ask if a client plays videogames as both an ice breaker and as part of the assessment. If a therapist is more knowledgeable about videogames or has even play videogames themselves, they may be able to better build rapport with a client provided they are mindful of their counter-transference more than a therapist that does not.

**Table 1:** **Demographical data obtained from the Entertainment Software Association (2015)**.

**Demographic**		**Percentage**
Game Players	Male	56
	Female	44
Age	Under 18	26
	18 to 35	30
	36 to 49	17
	Over 50	27
Game Purchasers	Male	59
	Female	41

*Data was obtained from the Entertainment Software Association (2015)*.

At a deeper level, videogames can enable therapists to gain meaningful insight about their clients. There are various videogame genres such as survival horror, role playing, and first person shooters and there is research available on some of these genres (i.e., Krzywinska, 2015; Younbo et al., 2015). Some videogames touch deeper themes such as funerals, death, and even trauma (Servais, 2015; Smethhurst, 2015). Krzywinska (2015) asserts that horror games provide a unique perspective not seen in other videogame genres. Krzywinska states that horror games can elicit complex and transformational forms of pleasure.

Younbo et al. (2015) looked at the effects of non-verbal sensitivity and gender on first-person shooter game experience. It was found that participants with high non-verbal sensitivity experienced more arousal than participants with low non-verbal sensitivity. They also found that biological sex became insignificant when taking non-verbal sensitivity into consideration.

By getting to know clients and the types of videogames they play, therapists can gain insight as to their clients' values, beliefs, and tastes. For example, a client whom enjoys playing horror games over other genres may be obtaining more complex forms of transformational pleasure than clients whom play first-person shooters. Conversely, clients whom play first-person shooters and have high non-verbal sensitivity can give a therapist more insight if a client reports high overall arousal levels.

Therapists can also integrate videogames into other therapeutic techniques. For example, this writer once conducted a session where this writer and the client were playing a racing videogame in session. The client commented that it reminded him of when he used to play videogames with his father. This writer asked the client what he would tell his father if he was also playing the racing game at that moment. The client talked about his sadness and said that he would tell his father that he misses him.

While there are several studies, as mentioned above, that look into the potential benefits of videogames in psychotherapy and counseling, more work needs to be done in disseminating these findings and integrating them into actual therapeutic practice. Therapists currently use art, movie clips, and educational books as therapeutic tools. There are mobile applications available for smartphones that resemble self-help educational books. Integrating the right genres of videogames as therapeutic tools in live sessions seems like a logical step in the process and can be useful due to their interactive nature if applied appropriately. Many, therapists and counselors are constantly working toward improving themselves and their practice. Perhaps it is time to use this increasingly popular medium of entertainment to enable clients to grow as individuals and achieve their therapeutic goals.

## Author contributions

The author confirms being the sole contributor of this work and approved it for publication.

### Conflict of interest statement

The author declares that the research was conducted in the absence of any commercial or financial relationships that could be construed as a potential conflict of interest.
